# Vitamin D deficiency in breastfed infants & the need for routine vitamin D supplementation

**Published:** 2011-03

**Authors:** S. Balasubramanian

**Affiliations:** Kanchi Kamakoti CHILDS Trust Hospital Chennai 600 034, India sbsped53@sify.com, sbsped@gmail.com

There has been increasing global interest regarding the role of vitamin D in health and disease. In fact, more and more scientific evidence linking vitamin D to various chronic diseases in children and adults is emerging. Prevention of vitamin D deficiency and achieving adequate intake of vitamin D and calcium throughout childhood may reduce the risk of osteoporosis as well as other long-latency disease processes that have been associated with vitamin D-deficiency states in adults^1^–^4^. Despite food fortification policies in many countries and recommendations for vitamin D supplementation of at-risk groups, vitamin D deficiency and infantile rickets remain major public health challenges in many developed and developing countries. There is evidence that the current supplementation recommendations, particularly for pregnant and lactating women, are inadequate to ensure vitamin D sufficiency in these groups.

Rickets attributable to vitamin D deficiency is known to be a condition that is preventable with adequate nutritional intake of vitamin D^5^. Rickets is an example of extreme vitamin D deficiency, with a peak incidence between 3 and 18 months of age. A state of deficiency occurs months before rickets is obvious on physical examination, and the deficiency state may also present with hypocalcemic seizures^6^, growth failure, lethargy, irritability, and a predisposition to respiratory infections during infancy^7^. Two types of presentation of vitamin D deficiency have been described in children^8^. The first was symptomatic hypocalcemia (including seizures) occurring during periods of rapid growth, with increased metabolic demands, long before any physical findings or radiologic evidence of vitamin D deficiency occurred. The second clinical presentation was that of a more chronic disease, with rickets and/or decreased bone mineralization and either normocalcemia or asymptomatic hypocalcemia.

Historically, the main source of vitamin D has been via synthesis in the skin from cholesterol after exposure to UV-B light. Full-body exposure during summer months for 10 to 15 min in an adult with lighter pigmentation will generate between 10000 and 20000 IU of vitamin D_3_ within 24 h; individuals with darker pigmentation require 5 to 10 times more exposure to generate similar amounts of vitamin D_3_^9^,^10^.

The amount of UV exposure available for the synthesis of vitamin D depends on many factors other than just time spent outdoors. These factors include the amount of skin pigmentation, body mass, degree of latitude, season, the amount of cloud cover, the extent of air pollution, the amount of skin exposed, and the extent of UV protection, including clothing and sunscreens^11^–^13^.

The multitude of factors that affect vitamin D synthesis by the skin^14^, the most important of which is degree of skin pigmentation, make it difficult to determine what is adequate sunshine exposure for any given infant or child^15^,^16^. It is still debated as to how much solar UV exposure is appropriate to balance between risks of vitamin D deficiency and skin cancer. This has given rise to the argument that sun avoidance, with a goal of skin cancer prevention, may compromise vitamin D sufficiency. Among dermatologists, there is active discussion about the risks and potential benefits of sun exposure and/or oral vitamin D supplementation · however, the vast majority would agree with the current American Academy of Pediatrics guidelines for decreasing sunlight exposure, which include the advice that infants younger than 6 months should be kept out of direct sunlight^17^.

## Pregnancy, vitamin D, and the foetus

A Cochrane review in 2002^18^ concluded that there are limited data available regarding maternal vitamin D requirements during pregnancy, despite the fact that maternal vitamin D concentrations largely determine the vitamin D status of the foetus and newborn infant. With restricted vitamin D intake and sunlight exposure, maternal deficiency may occur, as has been documented in a number of studies. It is important to note that women with increased skin pigmentation or who have little exposure of their skin to sunlight are at a greater risk of vitamin D deficiency and may need additional vitamin D supplements, especially during pregnancy and lactation^19^. Adequate nutritional vitamin D status during pregnancy is important for foetal skeletal development, tooth enamel formation, and perhaps general foetal growth and development^20^. There is some evidence that the vitamin D status of the mother has long-term effects on her infant.

These data suggest that doses exceeding 1000 IU of vitamin D per day are necessary to achieve 25-OH-D concentrations of >50 nmol/l in pregnant women^21^,^22^. The significance of these findings for those who care for the paediatric population is that when a woman who has vitamin D deficiency gives birth, her neonate also will be deficient.

Another study of the intrauterine effect of maternal vitamin D status revealed a significant association between umbilical cord 25-OH-D concentrations and head circumference at 3 and 6 months’ postnatal age that persisted after adjustment for confounding factors^23^. A United Kingdom study demonstrated that higher maternal vitamin D status during pregnancy was associated with improved bone-mineral content and bone mass in children at 9 yr of age^24^.

## Vitamin D deficiency and breastfeeding

Infants who are exclusively breastfed but who do not receive supplemental vitamin D or adequate sunlight exposure are at increased risk of developing vitamin D deficiency and/or rickets^25^,^26^. Infants with darker pigmentation are at greater risk of vitamin D deficiency, a fact explained by the greater risk of deficiency at birth^27^ and the decreased vitamin D content in milk from women who themselves are deficient.

Although vitamin D concentrations can be increased in milk of lactating women by using large vitamin D supplements, such high-dose supplementation studies in lactating women have not been validated and demonstrated to be safe in larger, more representative populations of women across various parts of the world. Recommendations to universally supplement breastfeeding mothers with high-dose vitamin D cannot be made at this time. Therefore, supplements given to the infant are necessary.

In this issue, Jain and colleagues from New Delhi^28^ have reported their significant observations on the prevalence of vitamin D deficiency and insufficiency [serum 25 hydroxyvitamin D (25OHD) < 15 ng/ml and 15-20 ng/ml, respectively] among healthy term breastfed 3 month-old infants and their mothers. At the present time, however, consensus has not been reached with regard to the concentration of 25OH-D to define vitamin D insufficiency for infants and children^29^. They have reported extremely high figures of vitamin D deficiency and insufficiency in the infants studied similar to earlier reports from other parts of India. Interestingly radiological rickets was also observed in their study amongst nearly one third of breastfed infants with vitamin D levels <10 ng/ml. Intake of vitamin supplement by the infant, sunlight exposure and maternal 25OH-D levels were found to have positive correlation with the infants’ 25OH-D. These findings suggest that hypovitaminosis D could be prevented by adopting maternal supplementation or increasing sunlight exposure for pregnant and lactating mother or by vitamin D supplementation to all infants routinely.

Although it is clear and incontrovertible that human milk is the best nutritive substance for infants during the first year, there has been concern about the adequacy of human milk in providing vitamin D. No one likes to attack breast milk and baby friendly paediatricians are concerned about the issues around ‘knocking’ breast milk and the suggestion that it is somehow inadequate to meet the needs of the newborn and young infants. However, it has been demonstrated over and over again that breast milk has very low levels of vitamin D which is significant for newborns with a vitamin D deficiency and for those who are exclusively breastfed for a prolonged period of time.

Considering the magnitude of the problem of hypovitaminosis D in infancy, there are several practical difficulties in ensuring adequate sunlight exposure to women and young infants which will involve massive health education campaigns. Added to this is the fear of increasing the risk of malignancies of the skin due to increase in ultraviolet radiation. Changing lifestyles, urbanization, cultural and religious beliefs limiting sunlight exposure due to covered clothing add to the difficulties in ensuring adequate sunlight exposure across populations. There is no consensus on the dosage of vitamin D that needs to be supplemented to pregnant women nor is there robust scientific evidence to support implementation of a vitamin D supplementation programme for pregnant and lactating mothers. The only practical option available is to seriously consider a routine vitamin D supplementation programme starting from neonatal period extending right through the childhood into adolescence. In a recently published study, oral vitamin D_3_ supplementation as an oil emulsion has been shown to be associated with significant and sustained increases in 25(OH)D from baseline in fully breastfeeding infants through 7 months^30^.
